# Hypofractionated Radiation Therapy for Large Brain Metastases

**DOI:** 10.3389/fonc.2018.00379

**Published:** 2018-10-02

**Authors:** Giuseppina Laura Masucci

**Affiliations:** Department of Radiation Oncology, Centre Hospitalier de l'Université de Montréal, Montreal, QC, Canada

**Keywords:** large brain metastasis, hypofractionation, stereotactic radiotherapy, radiosurgery, brain metastasis

## Abstract

Single fraction radiosurgery (SRS) treatment is an effective and recognized alternative to whole brain radiation for brain metastasis. However, SRS is not always possible, especially in tumors of a larger diameter where the administration of high dose in a single fraction is limited by the possibility of acute and late side effects and the dose to the surrounding organs at risk. Hypofractionated radiation therapy allows the delivery of high doses of radiation per fraction while minimizing adverse events, all the while maintaining good local control of lesions. The optimal dose fractionation has however not been established. This overwiew presents available evidence and rationale supporting usage of hypofractionated radiation therapy in the treatment of large brain metastases.

Brain metastases (BM) are a common occurrence in oncologic patients (1). Large BM can be defined according to their diameter or volume, with lesions measuring either ≥2 or ≥3 cm in diameter or ≥ 4 cm^3^ (2–8) being considered in this category. The optimal treatment for these tumors has not yet been established. The combination of surgery with post operative radiation either to the cavity or to the whole brain (WBRT), SRS alone or hypofractionated radiation therapy (HFRT) have been proposed to address these tumors (3–16) However, local control (LC) rates of large brain metastasis are known to be inferior to those of smaller dimension (4, 5, 14–20). When possible, surgery, with post operative radiation, should be considered (21) to decrease mass effect, alleviate neurological symptoms and facilitate management. For patients with large brain metastasis unable to undergo surgical resection, WBRT has been considered to be the standard of care. However, WBRT is associated with a poor local control for lesions of larger diameter (22). Nieder et al. (22) analyzed the efficacy of WBRT in controlling 336 brain metastasis in 108 patients. Local failure was estimated to be 48% in tumors measuring <0.5 cm^3^; however, all lesions measuring >10 cm^3^ recurred. Complete response was observed only in tumors measuring <6.4 cm^3^ although partial response was seen in large or necrotic metastases.

Radiosurgery (SRS) is increasingly becoming the preferred treatment for BM, not only for its efficacy in providing good local control, but also for its limited long term toxicity profile, especially regarding neurocognitive function when compared to whole brain radiation therapy (WBRT) (10, 23, 24). Moreover, the usage of SRS alone has not been linked to a decrease in OS (25). SRS alone is an effective treatment for smaller metastases. However, as tumor size increases, the dose that can be administered safely, without any neurological toxicity, decreases (26). In the dose escalation study RTOG 90-05 (27), lesions measuring ≤ 2, 2.1–3, and 3.1–4 cm were treated by radiosurgery with doses of 24, 18, and 15 Gy, respectively. By using this fractionation scheme, Vogelbaum et al. (20) reported that, while treatment with radiosurgery achieved only a LC of 49 and 45% in lesions measuring 2.1–3 cm in diameter and 3.1–4 cm, lesions measuring ≤ 2 cm achieved a LC of more than 85% when treated with a dose of 24 Gy. The same conclusions were made by Elliott et al. (28) and Schoeggl et al. (29) where the treatment of lesions measuring >10 and >17 mm, respectively by radiosurgery had more local failure. Petrovich et al. (30) concluded that 1 year LC of lesions <3 cc was greater (90%) when compared to that of lesions >3 cc (78%). Ebner et al. (15) concluded that lesions measuring ≥3 cm had a worse LC at 1 year (68%) then lesions <3 cm (86%). It has been speculated that better LC could possibly be achieved with a higher prescribed dose (18, 20, 24, 31). However, the administration of greater doses of radiation in one single fraction to a large volume is limited by the possibility of acute and late side effects and the dose to surrounding organs (OAR), for example the brainstem or optic nerves (32–34).

## Outcomes with hypofractionation for large metastasis

In an attempt to increase the biologically equivalent dose (BED) administered to BM and possibly LC while minimizing the risk of radiation induced toxicity, the administration of large doses of radiation in a few fractions (typically 2–6) has been studied (7, 12, 13, 16). Although this alternative to radiosurgery requires the patient to undergo multiple days of treatment, it has been associated with a median OS of 7–17 months and a 1 year LC of 64 to 100%. (3, 6, 7, 12, 13, 16, 35–42). In a review of 448 patients treated in eight series, it was concluded that HFRT can safely be administered in patients with lesions measuring >1 cm; furthermore, for tumors with a diameter >2 cm, HFRT seemed preferable to SRS, with LC of 68.2–93% and a low rate of radionecrosis of 3.1% (43).

Multiple studies have looked at the outcomes of patients treated with HFRT (Table 1). A prospective phase II study (36) evaluated the efficacy of HFRT in patients not amenable to SRS. Patients with lesions with a volume of >3 cc or located in eloquent area were considered. Median diameter of lesions treated was 2.27 cm. Seventy-two patients received 5 treatments of 6 Gy if WBRT was given or 5X 7 Gy in patients treated singlehandedly with HFRT. Complete response was seen in 66% of patients, possibly because of the median gross tumor volume (GTV volume) measured at 6 cc (0.29–65.57). Local control was deemed to be over 70% 1 year after treatment. Size of the treated volume was associated with a 7 months disease specific survival (DDSS) of 81% for tumors <6 cc vs. 53% for lesions ≥ 6 cc). Inoue et al. (40) looked at 88 patients treated with large BM measuring ≥10 cm^3^ (10–74.6 cm^3^). Tumors measuring 10–19.9 cm^3^ received 27–30 gy in three fractions (fx); the majority received 31–35 Gy in 5 fx for lesions 20–29.9 cm^3^ and 35–42 Gy in 8–10 fx was administered to those measuring ≥ 30 cm^3^. Median single dose equivalent of the maximum dose was 46–48 Gy. LC was seen in 90.2% of patients with no difference in LC, regardless of the volume treated. A study by Rajakesari et al. (41). retrospectively reviewed the outcomes of 112 patients treated with HFRT (87% received a dose of 25 Gy in 5 fx), 70 of which had brain metastasis measuring >3 cm. With a median follow up of 13.5 months, 1 year LC was 56%. Navarria et al. (7) treated 102 patients with HFRT. In this study, 27 Gy in 3 daily fx was administered to 51 brain metastasis measuring 2.1–3 cm; lesions of 3.1–5 c in diameter received 32 Gy in 4 daily fx. The fractionation was chosen to provide a biologically equivalent dose (BEDGY_10_) > 50 Gy. With these fractionation schemes, lesions, irrespective of the dose administered, had a 1 year LC of 96%.

**Table 1:** Selected series of patients treated with HFRT.

	**No of Pts/BM**	**Volume (cm^3^) diameter (cm) (median)**	**Median dose [prescribed isodose (%)]**	**BEDGy_10_**	**Median 1 year overall survival (OS) (months) (%)**	**1 year local control (LC) (%)**	**Radionecrosis (%)**
Feuvret et al. (16)	12 pts	29.4 c cm^3^ 4.4 cm	3X7.7Gy to PTV	39.4	504 days	100%	None
Fokas et al. (38)	102 pts	Gr 1: 2.04 cm^3^ Gr 2: 5.93 cm^3^	Gr.1(*n* = 61): 7X5gy Gr 2 (*n* = 61): 10X4Gy	Gr 1: 52.5 Gr 2: 56	Gr 1: 7 mo Gr 2: 10 mo	Gr 1: 75% Gr 2: 71%	1 patient in Gr 1
Inoue et al. (40)	88 pts/ 92 BM	16.2 c cm^3^	Gr 1: 10–19.9 cm^3^: 27–30 Gy in 3 fx Gr 2: 20–29.0 cm^3^: 31–35 Gy in 5 fx Gr 3: >30 cm^3^: 35–42 Gy in 8–10 fx (55–57%)	Gr 1: 51.3–60 Gr 2: 50.2–59.5 Gr 3: 50.7–59.6	9 mo	Marginal recurrences: GR 1: 7% Gr 2: 11% Gr 3: 0%	
Jiang et al. (13)	40 pts	17.5 cm^3^ 4.1 cm	40 gy (20–53) in 10 fx (4–15) isodose: 90% + boost 20 gy (10–35) in 4 fx (2–10) in 23 patients 1–3 months after tx	56 + 30	15 mo 55.3%	94%	None
Minniti et al. (12)	138 pts	12.5 cm^3^	27 Gy in 3 fx (80–90%)	51.3	13.4 months 56%	90% (for lesions ≥3 cm 73%)	9% for HFRT 14% for lesions >3 cm
Navarria et al. (7)	102 pts 51 Gr 1 51 Gr 2	16.3 cm^3^ 2.9 cm	Gr 1: diameter 2.1–3 cm: 27 Gy in 3 fx Gr 2: diameter 3.1–5 cm: 32 Gy in 4 fx (80%)	Gr 1: 51.3 Gr 2: 57.6	14 mo 69% Gr 1: 14 mo 60% Gr 2:14 mo 80%	96% Gr 1: 100% Gr 2:91%	5.8%
Murai et al. (6)	54 pts/ 61 BM	≥2.5 cm	diameter 2.5–3 cm: 3 fx diameter ≥4 cm: 5 fx Gr 1: 18–22 Gy in 3 fx 21–25 Gy in 5 fx Gr 2: 22–27 Gy in 3 fx 25–31 Gy in 5 fx Gr 3: 27–30 Gy in 3 fx 31–35 Gy in 5 fx	Gr 1: 28.8–39.4 29.8–37.5 Gr 2: 39–51.3 37.5–50.2 Gr 3: 51.3–60 50.2–59.5	31%	69% Gr1: 66% Gr 2: 65% Gr 3: 68%	None
Rajakesari et al. (41)	70 pts	1.7 cm	25 Gy in 5 fx (90–95%)	37.5	10.7 mo	56%	4.3%
Fahrig et al. (37)	150 pts/ 228 BM Gr 1: 72 Gr 2: 59 GR 3: 97	6.1 cm^3^	Gr 1: 5X 6–7Gy Gr 2: 10 X 4 Gy Gr 3: 7X 5Gy (90%)	Gr 1: 48–59.5 Gy Gr 2: 56 Gy GR 3: 52.5 Gy	16 mo 83% Gr 2 and Gr 3: 17 mo Gr 1: 11 mo	Gr 1: 87% Gr 2: 95% GR 3: 96%	1.3%
Aoyama et al. (44)	87 pts/ 159 BM		35 Gy in 4 fx	62.9	8.7 mo	81%	
Ernst-Stecken et al. (36)	51 pts/ 72 BM	2.27 cm 6 cm^3^	Gr 1: If WBRT prior: 5X 6Gy Gr 2: no WBRT: 5X 7Gy (90%)	Gr 1: 58 Gr2: 59.5	11 mo	76%	2%
**MULTIPLE STAGE RADIOSURGERY SERIES**							
Higuchi et al. (39)	43 pts	17.8 cm^3^	10 Gy in 3 fx, 2 weeks apart	60	8.8 mo 76.2%	75.9%	None
Yomo and Hayashi (42)	58 pts	16.4 cm^3^	20–30 Gy in 2 fx; 3–4 weeks apart (45%)	40–75	11.8 mo 47%	64%	None
Angelov et al. (3)	54 pts/ 63 BM	10.54 cm^3^ 3.3 cm	30 Gy in 2 fx 1 months apart (54%)	75	10.8 49%	88% (@ 6 mo)	3.17%
Dohm et al. (35)	33 pts/ 39 BM	11.68 cm^3^	15 Gy in 1 fx followed a month later by 14 Gy in 1 fx (50%)	37.5–33.6	60%	87%	10.2%

### Srs vs. hypofx

Feuvret et al. (16) published the outcomes of 36 patients treated for solitary BM larger than 3 cm in diameter (median diameter 3.7 cm), with either radiosurgery or HFRT. Patients in this case series received either 14 Gy in one fraction or 3 fractions of 7.7 Gy. One year LC rates differed between the two cohorts, with 100% of lesions treated with HFRT being controlled vs. 58% in patients treated with SRS. Moreover, no cases of radionecrosis were reported. Minniti et al. (12) confirmed these results in a retrospective study of patients treated with BM measuring >2 cm. A HFRT treatment of 27 Gy in 3 fx was compared to a SRS in which tumors measuring 2–3 cm received 18 Gy and lesions measuring ≥3 cm 15–16 Gy. One year LC rates were statistically different between the two groups, with 90% of patients treated with HFRT vs. 77% of patients treated with SRS attaining LC at 1 year (12).

## Factors influencing local control and overall survival after HFRT for large metastases

Multiples prognostic factors have been analyzed to assess OS and LC of brain metastases treated with HFRT (Table 2). However, none of the studied factors were predictive of OS or LC by all authors. Patient overall well-being, identified with the Karnofsky Performance Score (KPS) as well as the patient's recursive partitioning analysis (RPA) score seem to be predictive of overall survival in a number of studies (3, 7, 12, 13, 36–38). Local control seems to be influenced by the dose administered and the size of the treated tumor, albeit not by all.

**Table 2:** Selected series with factors influencing OS and local control in patient treated with HFRT.

	**Overall survival**		**Local control**	
	**SS**	**NS**	**SS**	**NS**
Ebner et al. (15)	**UVA:** Age (<65 years) Controlled primary **MVA:** Age <65 years	**Size:** <3.0 cm vs. ≥3 cm		**UVA:** Gender Age ≥65 Histology Surgical resection status Dose (≤16 vs. >16 Gy) Tumor size: 3–4 cm vs. ≥4 cm
Inoue et al. (40)	Lower survival for lesions ≥ 30 cm^3^			**On UVA and MVA:** Age Gender Tumor location within brain Tumor volume Number of fraction of RT V14 Tumor size: 10–19.9 cm^3^ vs. 20–29.9 cm^3^ vs. ≥ 30 cm^3^
Fokas et al. (38)	**UVA:** Chemotherapy status (yes vs. no) RPA class: I vs. II-III Single BM (vs. multiple BM) Presence of extracerebral disease **MVA**: RPA class I	Surgical resection status Age Gender	Dose administered (srs vs. 7X 5gy vs. 10X 4gy)	
Jiang et al. (13)	Controlled primary tumor KPS≥ 80	Gender Age Number of brain mets presence of extracranial disease RPA class		Gender Age number of brain mets presence of extracranial metastasis KPS RPA class
Minniti et al. (12)	**MVA:** Extracranial disease (stable) Histology: breast cancer (better) KPS >70		Histology: melanoma worse local control	No other actors were predictive of local failure; Tumor size > 3 cm was of borderline significance
Navarria et al. (7)	**UVA and MVA:** KPS Extracranial disease (stable)		**UVA and MVA:** Gender Age KPS Histology Presence of extracranial disease RPA-GPA class Tumor size	
Yomo and Hayashi (42)	Extracranial disease (stable) Interval from cancer diagnosis to RT treatment (<12 vs. > 12 months) Single vs. multiple BM	Age (≤65 vs. >65) KPS ≥90		
Fahrig et al. (37)	**MVA:** RPA class	**MVA:** RT dose (5 X 7Gy vs. 10X 4Gy vs. 7 X5gy)	Trend for better LC for lesions treated with 5X6-7Gy and 7X5Gy vs. 10X4Gy	
Ernst-Stecken et al. (36)	Tumor size KPS Number of metastases (1 vs. >1)	Extracranial disease Age (≤65 vs. >65) Gender		
Angelov et al. (3)	**UVA:** Interval from cancer diagnosis to RT treatment (<12 vs. > 12 months) KPS (<70) Number of lesions <2 cm Greater volume of tumor present at second hypofx treatment (≤3.5 vs. >3.5 cm^3^) **MVA**: KPS Number of lesions <2 cm greater volume of tumor present at second hypofx treatment (≤3.5 vs. >3.5 cm^3^)		**UVA:** Volume change between first and second hypofx treatment KPS **MVA:** Volume change between first and second hypofx treatment	

*SS, statistically significant; NS, non-statistically significant; RPA, recursive partitioning analysis; KPS, Karnofsky Performance Score; GPA, graded prognostic assessment; UVA, univariate analysis; MVA, multivariate analysis; BM, brain metastasis*.

## Multiple stages stereotactic radiosurgery

A possible alternative to single fraction SRS and hypofractionation for large brain metastasis is a planned multiple treatment radiosurgery over two or more sessions separated by weeks or months (3, 35, 39, 42). Higuchi et al. (39) published in 2009, a study involving 43 patients treated for BM measuring ≥10 cm^3^ with 30 Gy delivered in 3 fx every 2 weeks. After delivery of 10 and 20 Gy, a reduction in volume of 18.8% and almost 40%, respectively, was noted in more than 90% of tumors. A 12 months LC of 75.9% was reported. Yomo and Hayashi (42) used a two stage treatment with radiation administered every 3–4 weeks. Fifty-eight BM with a volume of >10 cc were treated with a total of 20–30 Gy. One year LC of 64% was observed. Angelov et al. (3) reported results from 54 patients treated for 63 BM ≥2 cm in diameter with a total dose of 24–33 Gy (median 30 Gy) (BEDGy_10_: 44–73; median 62.5 Gy) in 2–3 fx to the target. Time between the first and second treatment was 1 month. Tumors were usually replanned before each treatment and volumes redefined. Analogous to the results published by Higuchi, they noted a median decrease in tumor volume of 17%; 90% of the lesions showed no progression with 67% of lesions showing a decrease in volume of ≥30 and 24% remaining stable. At 6 months follow up, LC was 88%. Dohm et al. (35) reported the results of 33 patients treated for 39 lesions in 2 treatments separated by 4 weeks. A median dose of 15 Gy (10–21 Gy) and 14 Gy (10–18 Gy) were administered on first and second treatment, respectively. One year local failure was 13%. Median volume reduction after first treatment was 32.6% and was observed in 33 tumors.

## Dose to target volumes and organs at risk (OAR)

In the treatment of BM with a single radiosurgery treatment, most radiation oncologists will prescribe doses in keeping with RTOG 90-05 (26); larger brain metastases, with a diameter of 3–4 cm, would therefore receive a single dose of 15 Gy. However, for these tumors, LC rates at 12 months are suboptimal, ranging from 37 to 62% (18, 26, 27). Vogelbaum et al. (20) published results from more than 200 patients that received radiosurgery in a single fraction. Although the results were similar to the ones previously stated, LC was deemed to be 45–49% when lesions received 15–18 Gy, but increased to 85% when 24 Gy was administered. However, doses of 24 Gy have been associated with a higher risk of CNS toxicity, of which the most feared is radionecrosis (26).

One of the advantages of hypofractionation is the delivery of a higher BED while minimizing the risk of side effects to the surrounding OAR. Nevertheless, the optimal dose to administer is not known. In the literature, multiple fractionation schemes have been studied (Table 1). Most use a minimum of 4 Gy and a maximum of 10 Gy per fraction. A total BEDGy_10_ of at least 50 Gy seems to provide better local control (38). Marcrom et al. (45) compared a dose of 25 Gy in 5 fx to 30 Gy in 5 fx in 72 patients treated for 182 BM measuring up to 5.5 cm (39 cc); 36 lesions being ≥3 cm in diameter. A total dose of 30 Gy was associated with a better LC 1 year after treatment (72 vs. 40% for lesions receiving 25 Gy). Fahrig et al. (37) assessed three different doses to BM with a maximal diameter >3 cm. Patients received either 5 fx of 6–7 Gy (total: 30–35 Gy) in group 1, 10 fx of 4 Gy (total 40 Gy) in group 2 or 7 fx of 5 Gy (total 35 Gy) in group 3. Of these three regiments, the last two seemed to provide better 1 year LC and median OS when compared to group 1. This difference in OS between the three groups could possibly be explained by the fact that there were significantly less patients with RPA class I in group 1. CNS toxicity was deemed to be lesser for patients in group 2. On the other hand, a dose escalation study administering doses ranging from 18–22 Gy in 3 fx to 31–35 Gy in 5 fx did not demonstrate any difference in local control or overall survival in patients (6).

### Dose to OAR

Although the optimal doses to be administered to the brain metastasis are not known, dose constraints to be applied to nearby critical organs (OAR) are less controversial. Maximum doses have been limited to 21–25 Gy in 5 fx or 15–18 Gy in 3 fx (40, 45, 46) for the optical apparatus and to 31 Gy in 5 fx or 23 Gy in 3 fx for the brainstem (45, 46). Other possible dose limits that have been described for the brainstem are D1% (dose administered to 1% of the volume) ≤ 20 Gy or V26Gy (volume of the brainstem receiving 26 Gy) <1 cc, D1% ≤ 15 Gy or V20Gy (volume receiving 20 Gy) <0.2 cc for the optical nerves and D1% <1 Gy for the lenses (7, 45). Maintaining a V14Gy <3 cm^3^ for the brain parenchyma and <1 cm^3^ for critical areas such as motor cortex, basal ganglia or thalamus has been described (40).

## Post operative treatment of large cavities

### Cyst aspiration

Tumor size can influence the local control of brain metastases and overall survival of patients as stated above. It can therefore be of interest to reduce their volume prior to radiation treatment, permitting the administration of a higher radiation dose. An option for size reduction of cystic lesions is cyst aspiration, where a substantial decrease in tumor volume has been reported (47–50) (50.8–77.9%). This could potentially allow for treatment with a higher radiation dose (48). By combining this method to adjuvant radiation, better local control can be obtained, ranging from 45.8 to 63% (47–50). The latter also allows for the relief of acute symptoms related to mass effect (51, 52).

### Surgical resection

As previously mentioned, surgery should be considered for the treatment of large brain metastases. Post operatively, cavities can easily have a diameter > 3–4 cm, rendering a radiosurgery treatment difficult. Larger cavities are thus usually treated with a hypofractionated treatment with doses ranging from 24 Gy in 3 fractions to 36 Gy in 6 fractions (53–56). Most studies published have used a planning tumor volume (PTV) of 2–3 mm (57–59). With most failures occurring within the surgical cavities (60), a PTV margin of 2–3 mm seems to be sufficient.

The treatment of surgical cavities with fractionated radiation confers good local control, ranging from 77 to 93% (2, 12, 54, 61, 62) in the literature. Moreover, local control of larger cavities does not appear to be associated with the number of fractions or dose used (63). Histology of the primary, does not seem to influence recurrence, with similar local control for radiosensitive (i.e., breast and lung up to 94%) and radioresistant tumors [up to 90% i.e., melanoma, renal cell carcinoma (2, 12)] reported. Median survival after surgery and hypofractionated radiation treatment to cavities of large metastasis is 5.5–17 months (2, 11, 12, 60, 61, 64). A possible advantage of WBRT over HFRT in the post operative setting is the risk of leptomeningeal disease. The rate of leptomeningeal spread to meninges and cerebrospinal fluid in patients treated with WBRT is 5–12% (65, 66) vs. 14–28% (66, 67).

## Adverse effects

In the setting of hypofractionation, the rate of radiation necrosis has been estimated to be up to 10–15% (3, 6, 7, 11, 12, 16, 35–42). Authors have tried to determine dosimetric parameters and tumor characteristics that could possibly predict the risk of radionecrosis and severe CNS toxicity. In series comparing the usage of SRS vs. HFRT for the treatment of metastases, the rate of radionecrosis seems to be higher when patients are treated with a single fraction. Data (12) has showed that large tumors treated with 9 Gy in 3 fx had a 14% risk of radionecrosis vs. 33% for lesions treated in a single fraction. The risk of RN when treated with 3 fractions seems to be related to the volume receiving 18 Gy (12). Rates of radionecrosis are estimated to be 5% for V18 ≤ 30.2 cm^3^ and up to 14% for V18 > 30 cm^3^ (12). When analyzed according to quartile distribution, the risk was estimated to be: 0, 6, 13, and 24% for V18 <22.8, 22.8–30.2, 30.3–41.2, and >41.2 cm^3^, respectively (12). Others, Inoue et al. (40) have found that the surrounding brain volume treated to the equivalent of a single dose of 14 Gy (V14Gy) can be predictive of the risk of radionecrosis, with V14 ≥ 7.0 cm^3^ being a risk factor for developing extensive brain oedema and RN. It has been concluded that the risk of RN can be maintained under 2–15% when a BED of 90–127 Gy3 (α/β = 3) is used (dose of 24–35 Gy in 3–5 fx) (12, 36). Size has also been reported as a possible culprit, however inconsistently, with lesions of >3 cm at a higher risk (45).

Neurological symptoms related to HFRT has been reported in patients necessitating long term steroid treatment (13, 35, 36). Deaths secondary to surrounding oedema and the presence of radionecrosis, although rare, have also been described (13). Toxicity of lesser severity (grade 1–3) (according to the *National Cancer Institute Common Terminology Criteria for Adverse Events v.3 and v.4*) has been reported in 2–52% (12, 16, 36, 38) of patients treated with HFRT. Age (>60), treatment with less than five fractions, and a greater treated volume (possibly of >20 cm^3^) (36, 40) have been suggested to be predictive of brain oedema necessitating steroids. Lesions located deep within the white matter are perhaps more likely to cause oedema necessitating corticosteroids, and it has been suggested for these to keep V14Gy to ≤ 3 cm^3^ (40).

## Planning for radiation therapy

Planning of a hypofractionated radiation treatment for large brain metastases is very similar to that of a radiosurgery treatment. Patients usually undergo a planning CT and a high-field 3D distortion corrected T1 contrast MRI with isotropic voxels ≤ 1 m MRI with gadolinium to help delineate tumor volumes. Gross target volume (GTV) is delineated on CT scan and MRI and is defined as the area of contrast enhancement. Clinical target volume (CTV) is usually not defined in the treatment of brain metastasis treated with upfront radiation. However, in post operative treatment, it is defined as any contrast enhancing post operative changes on planning MRI and does usually not include the surgical tract (65, 68). In both situations, surrounding oedema is usually not included in treatment volumes. Planning tumor volume (PTV) is defined by adding a geometric margin of 1–3 mm (6, 7, 16, 24, 36). Treatment can be administered using different delivery systems and is usually linear-accelerator based to avoid head frame fixation as patients are usually treated with multiple fractions. However, treatments with dedicated intracranial radiosurgery unit such as the *Gamma Knife* have been published, especially in the setting for multi-staged treatment administered weeks apart (3, 42).Treatments can be delivered using multiple conformal arcs, static field IMRT or a dedicated radiosurgery unit such as *CyberKnife*®. As with any high dose per fraction treatment, image guidance is a must and has to be performed daily for patient set up and positioning verification.

## Conclusions

Hypofractionated radiation therapy treatment is a viable alternative to WBRT for the upfront treatment of brain metastasis that are not amenable to radiosurgery or surgery, or in the postoperative setting. It is associated with an accepted toxicity profile and good local control of lesions. The optimal dose fractionation is however still unknown and necessitates further investigation.

## Author contributions

The author confirms being the sole contributor of this work and has approved it for publication.

### Conflict of interest statement

The author declares that the research was conducted in the absence of any commercial or financial relationships that could be construed as a potential conflict of interest.
